# Supplementary material for: Single-cell transcriptomics identifies the differentiation trajectory from inflammatory monocytes to pro-resolving macrophages in a mouse skin allergy model
Source: Nat Commun. 2024 Feb 23;15:1666. doi: 10.1038/s41467-024-46148-4 (PMC10891131; doi:10.1038/s41467-024-46148-4)
Supplement: Supplementary file 1 — Supplementary Information [file 41467_2024_46148_MOESM1_ESM.pdf]

Supplementary Figures:

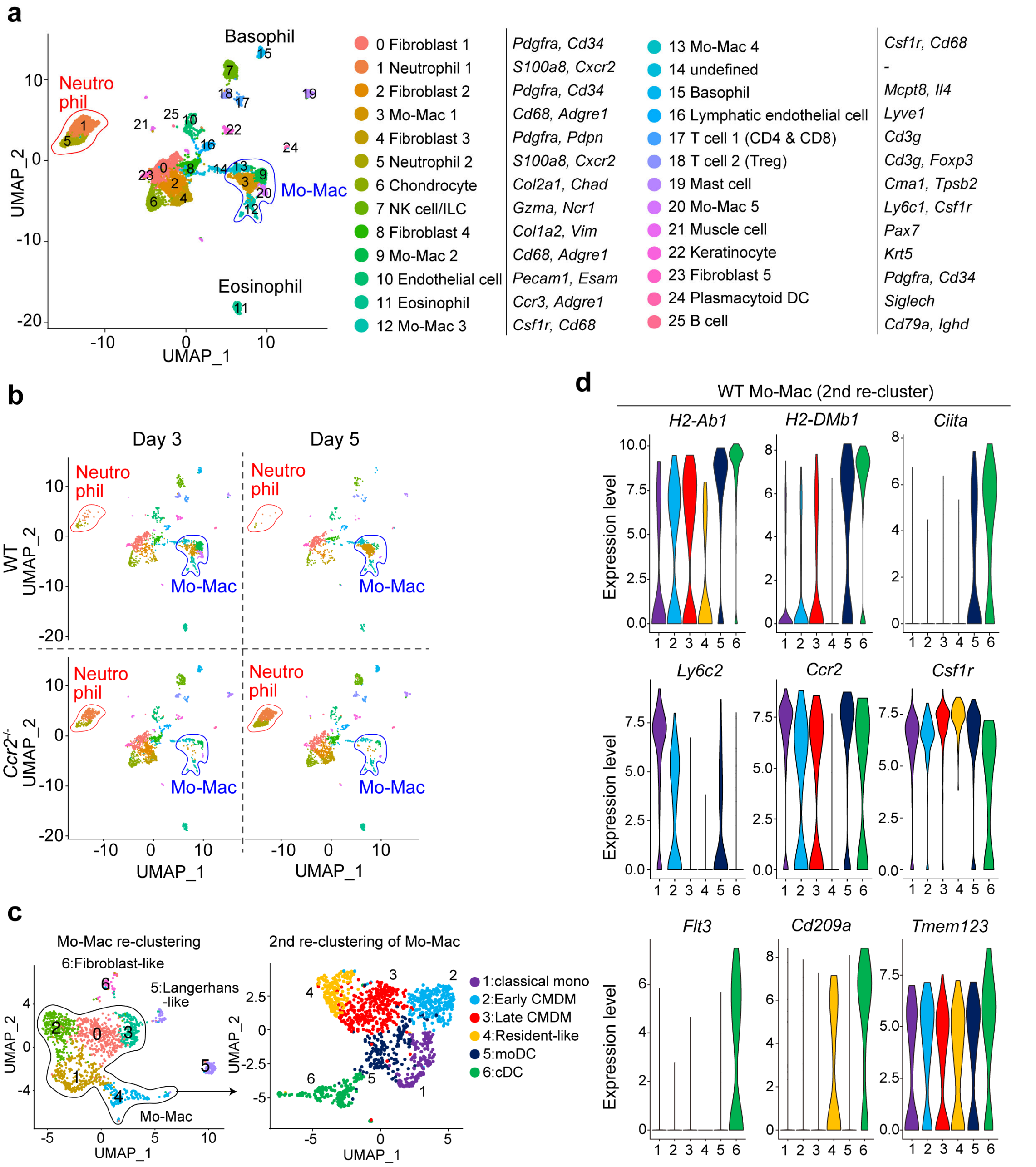

Supplementary Fig. 1. **scRNA-seq analysis identifies various cell lineages present in the IgE-CAI skin lesion.** IgE-CAI was elicited in WT and *Ccr2*$^{-/-}$ mice, and cells isolated from the skin lesion on days 3 and 5 post-challenge were subjected to scRNA-seq analysis. (a) UMAP plot of combined scRNA-seq datasets obtained from WT and *Ccr2*-/- mice is shown. (b) UMAP plots of total cells isolated from WT and *Ccr2*$^{-/-}$ mice on days 3 and 5 post-challenge are separately shown. (c) Monocyte-macrophage (Mo-Mac) clusters (clusters 3, 9, 12, 13, 20 shown in a were re-clustered. UMAP plot of re-clustered Mo-Mac populations are shown in the left panel. Mo-Mac lineage clusters (clusters 0, 1, 2, 3,

4) were further re-clustered for further analysis. UMAP plot of 2nd re-clustered Mo-Mac populations is shown in the right panel. (d) Violin plots of indicated gene expression in 2nd re-clustered Mo-Mac clusters are shown.

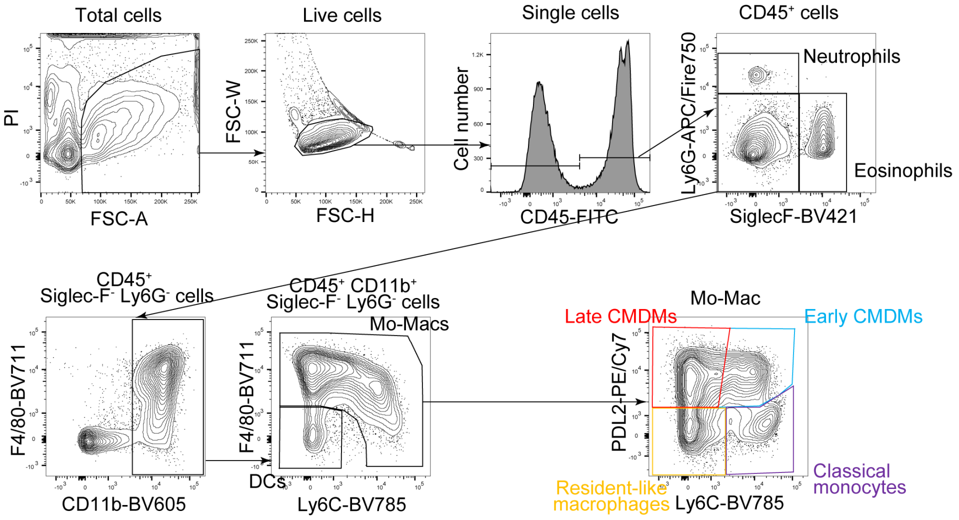

Supplementary Fig. 2. Gating strategy to identify each monocyte-macrophage subset. Gating strategy in flow cytometric analysis to identify monocytes, early CMDMs, late CMDMs and resident-like macrophages among the monocyte-macrophage cell lineage in the IgE-CAI skin lesion isolated 5 days after challenge. Data shown are representative of three independent experiments.

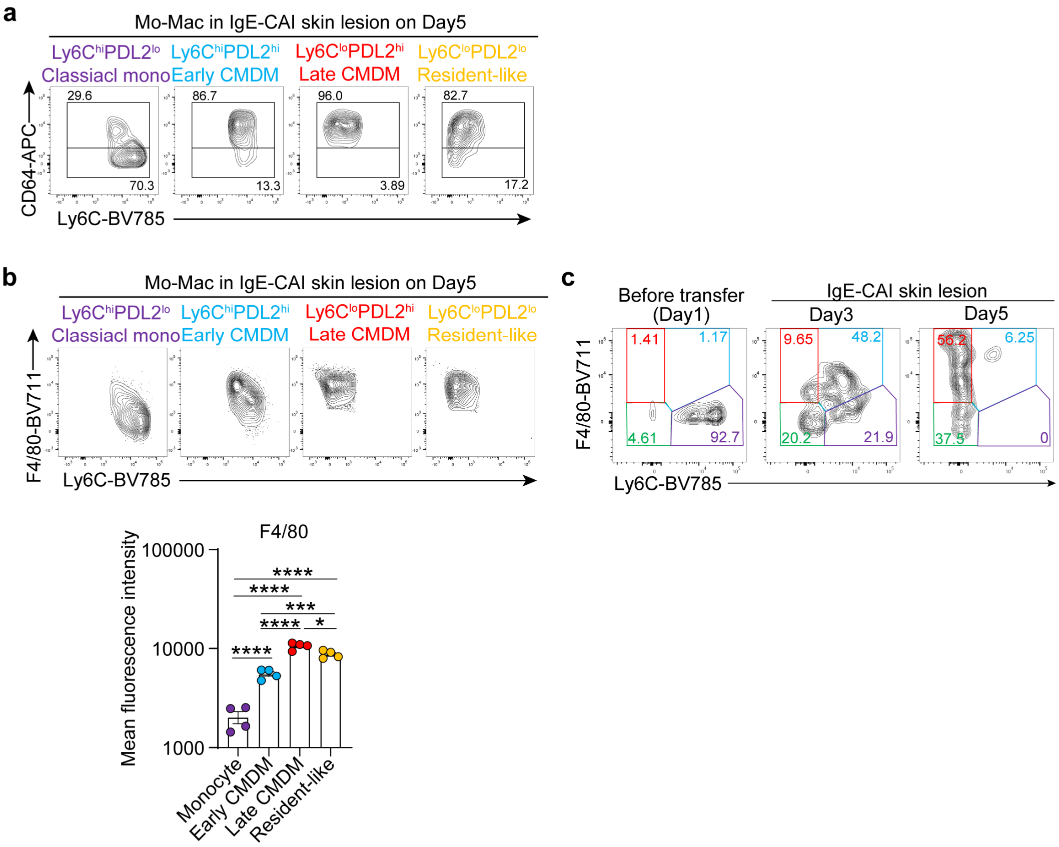

Supplementary Fig. 3. **Classical monocytes upregulate their CD64 and F4/80 expression along with the differentiation toward CMDMs.** (a-b) WT mice were treated as in Fig. 1 to induce IgE-CAI. Cells isolated from the IgE-CAI skin lesion on day 5 were subjected to flow cytometric analysis. In a, the surface expression of CD64 and Ly6C in $Ly6C^{hi}PDL2^{lo}$, $Ly6C^{hi}PDL2^{hi}$, $Ly6C^{lo}PDL2^{hi}$ and $Ly6C^{lo}PDL2^{lo}$ populations is shown. In b, the surface expression of F4/80 and Ly6C in $Ly6C^{hi}PDL2^{lo}$, $Ly6C^{hi}PDL2^{hi}$, $Ly6C^{lo}PDL2^{hi}$ and $Ly6C^{lo}PDL2^{lo}$ populations is shown in upper panels. The mean fluorescence intensity (MFI) of F4/80 in each Mo-Mac subset isolated from the ear skin on day 5 post-challenge is shown (mean ± SEM, n = 4 biologically independent animals for each group). ****p=9.58×10^{-5} (mono vs. early CMDM); ****p=6.1×10^{-9} (mono vs. late CMDM), ****p=9.47×10^{-8} (mono vs. resident-like), ****p=2.07×10^{-6} (early vs. late CMDM), ***p=0.0002 (early CMDM vs. resident-like), *p=0.0162 (late CMDM vs. resident-like) measured by one-way ANOVA with Tukey's multiple comparison test. (c) $Ly6C^{hi}$ monocytes prepared from the bone marrow of $CD45.1^{+}$ mice were adoptively transferred to $CD45.2^{+}$*Ccr2*-/- mice on day 1 post-challenge. $CD45.1^{+}$ cells isolated from the IgE-CAI skin lesion on days 3 and 5 were subjected to flow cytometric analysis. The surface expression of F4/80 and Ly6C in monocyte-macrophage lineage cells is shown.

Data shown in a-b are representative of three independent experiments. Data shown in c are representative of two independent experiments. Source data are provided as a Source Data file.

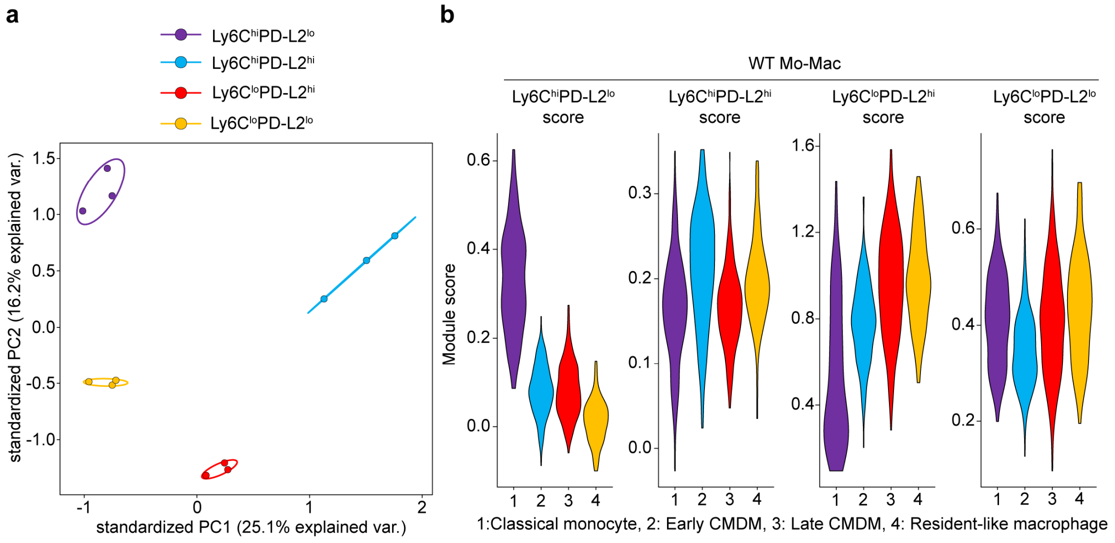

Supplementary Fig. 4. **Bulk RNA-seq analysis of individual Mo-Mac populations validates the correspondence to the Mo-Mac clusters identified in the scRNA-seq analysis.** WT mice were treated as in Fig. 1 to induce IgE-CAI. Four Mo-Mac populations (Ly6C^{hi}PDL2^{lo}, Ly6C^{hi}PDL2^{hi}, Ly6C^{lo}PDL2^{hi} and Ly6C^{lo}PDL2^{lo}) were isolated from the IgE-CAI skin lesion on day 5 and subjected to bulk RNA-seq analysis (n=3 samples, each). (a) PCA plot for the gene expression of four Mo-Mac populations is shown. (b) Differentially expressed genes (DEGs) in each Mo-Mac population were calculated by using bulk RNA-seq data. Module scores were calculated for scRNA-seq dataset in Fig. 1f concerning the DEGs for each Mo-Mac population. Violin plots showing the scoring for each Mo-Mac population are shown.

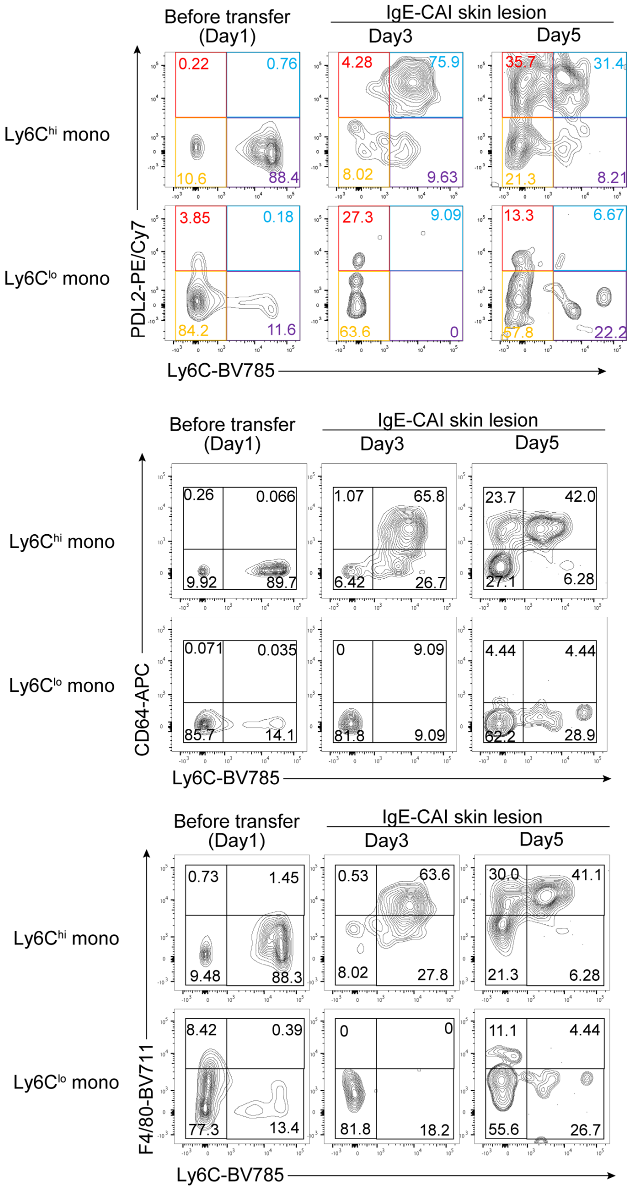

Supplementary Fig. 5. Classical but not non-classical monocytes sequentially differentiate into early and late CMDMs. WT mice were treated as in Fig. 1 to induce IgE-CAI. Ly6C^{hi} and Ly6C^{lo}monocytes isolated from the bone marrow of CD45.1^{+} mice were adoptively transferred to WT mice (CD45.2^{+}) on day 1 post-challenge. Cells isolated from the IgE-CAI skin lesion on days 3 and 5 were subjected to flow-cytometric analysis. The surface expression of PD-L2 and Ly6C (upper panels), CD64 and Ly6C (middle panels) or F4/80 and Ly6C (lower panels) in the CD45.1^{+} Mo-Mac population is shown. Data shown are representative of two independent experiments.

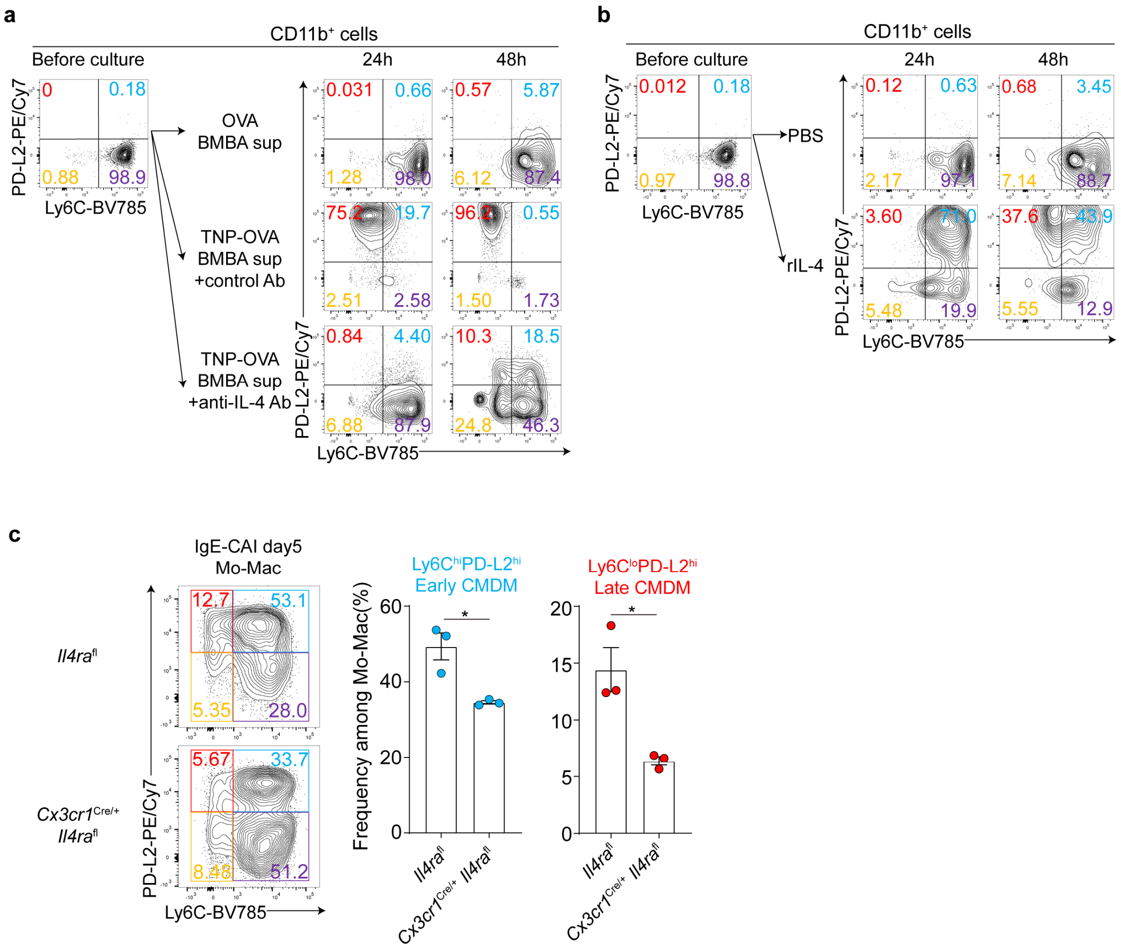

Supplementary Fig. 6. Classical monocytes differentiate into early and late CMDMs in an IL-4/IL-4 receptor-dependent manner. (a) Ly6C^{hi} classical monocytes isolated from the bone marrow of WT mice were incubated *ex vivo* with BMBA supernatants in the presence of anti-IL-4 blocking antibody or its isotype-matched control antibody. The surface expression of Ly6C and PD-L2 in monocytes before the culture (left) and after 24hr- or 48hr-incubation with BMBA supernatants (middle and right, respectively) are shown. (b) Ly6C^{hi} classical monocytes isolated from the bone marrow of WT mice were incubated *ex vivo* with recombinant mouse IL-4 (20ng/mL) or control PBS. The surface expression of Ly6C and PD-L2 in monocytes before the culture (left) and after 24hr- or 48hr-incubation with BMBA supernatants (middle and right, respectively) are shown. (c) *Cx3cr1*$^{Cre/+}$ *Il4ra*^{fl} or control *Il4ra*^{fl} mice were treated as in Fig. 1 to induce IgE-CAI. Cells isolated from the IgE-CAI skin lesion on day 5 were subjected to flow cytometric analysis. The surface expression of PD-L2 and Ly6C in Mo-Mac populations were shown in left panel. The frequency of Ly6C^{hi}PDL2^{hi} early CMDMs or Ly6C^{hi}PDL2^{lo} late CMDMs among Mo-Mac populations were shown in the middle and right panels (n=3

biologically independent animals for each group, mean±SEM). *p=0.0146 (middle panel) and * p=0.015 (right panel) measured by two-sided unpaired Student's t test. Data shown in a-b are representative of three independent experiments. Data shown in c are representative of two independent experiments. Source data are provided as a Source Data file.

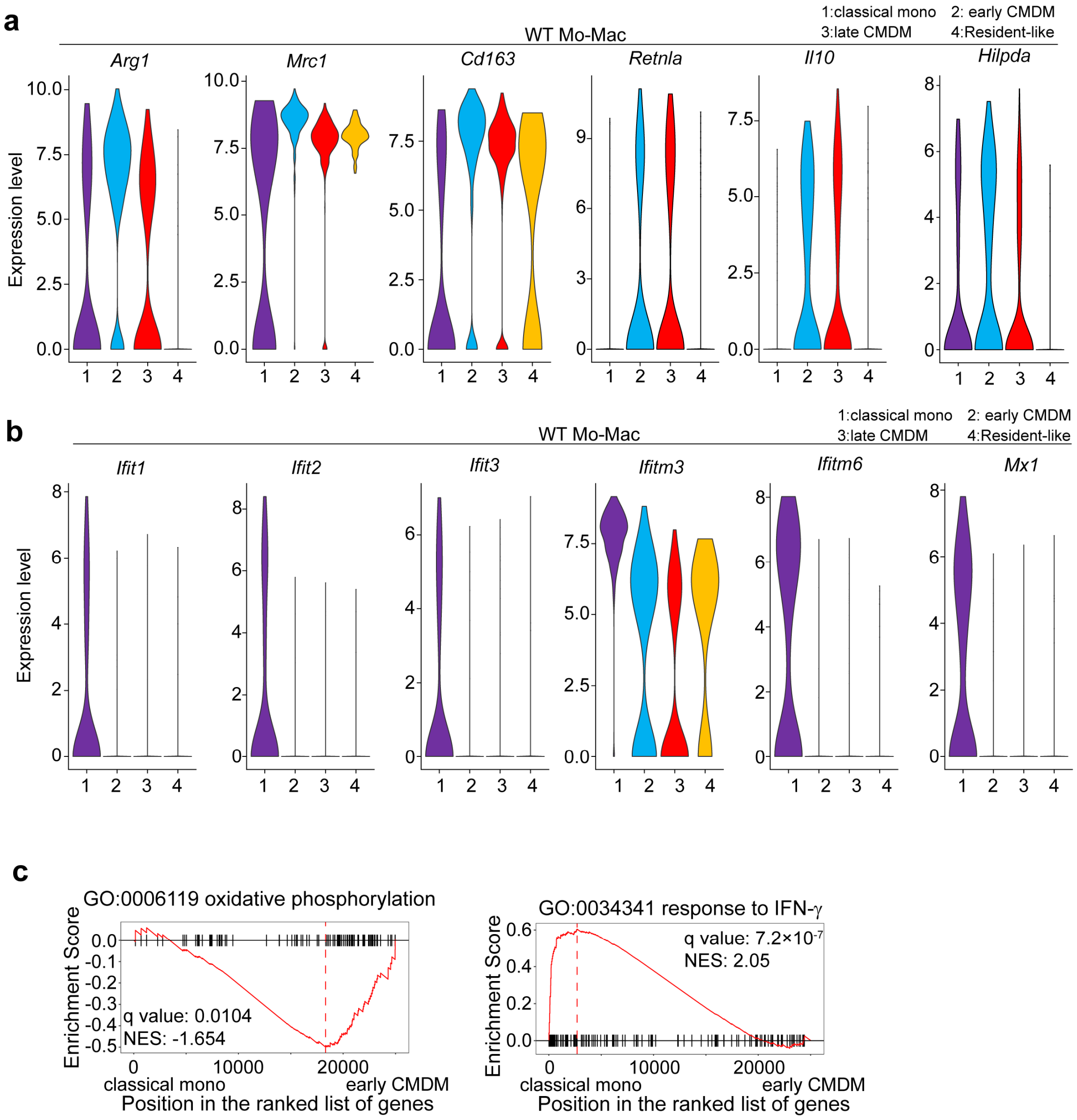

Supplementary Fig. 7. Early CMDMs show upregulated expression of anti-inflammatory genes and downregulated expression of interferon-inducible genes. (**a**-**b**) Violin plots of indicated gene expression in Mo-Mac clusters 1-4 are shown. (**c**) Early and late CMDMs were compared by using GSEA enrichment plot of genes involved in oxidative phosphorylation (left) and response to IFNγ (right).

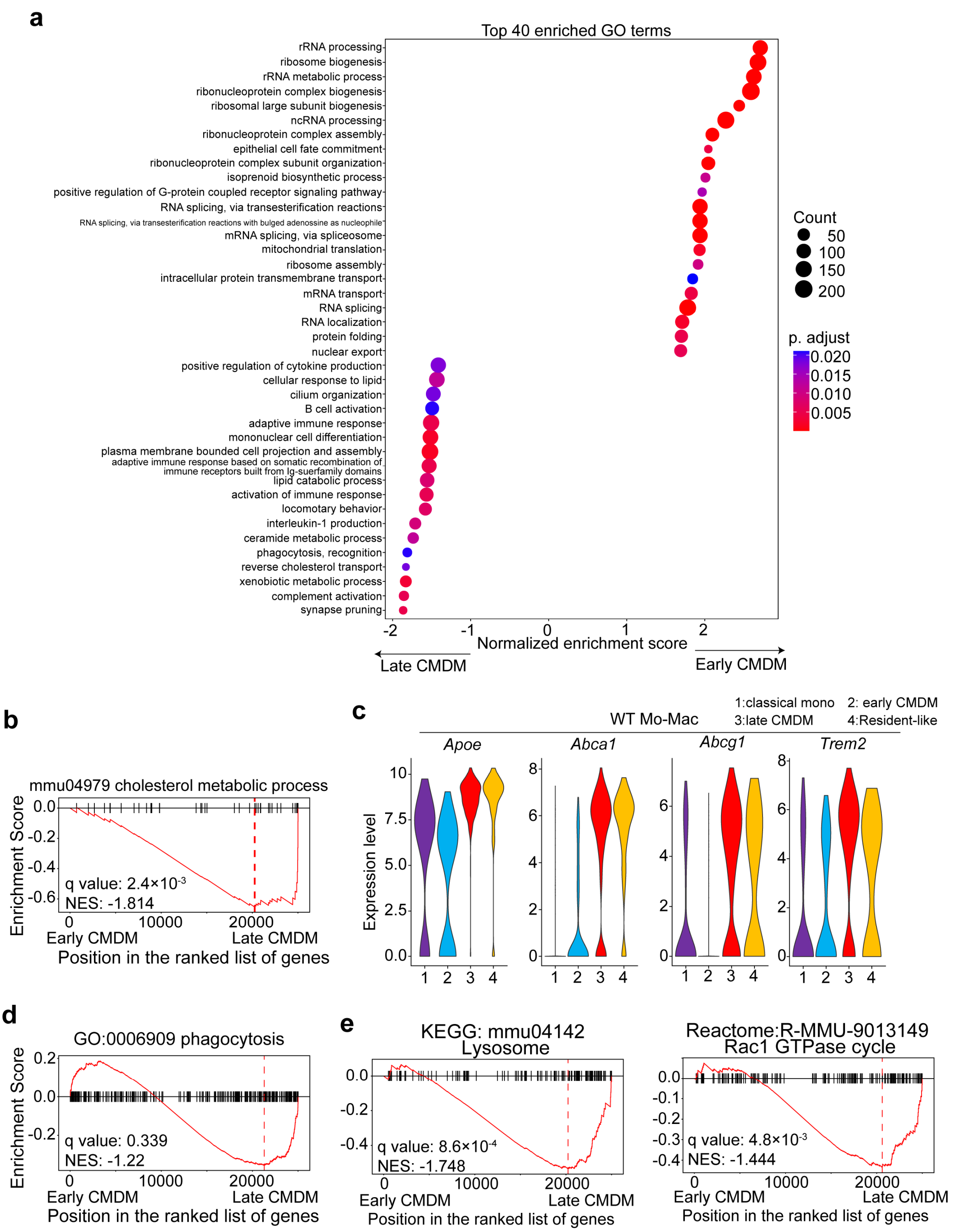

Supplementary Fig. 8. Late CMDMs display upregulated expression of phagocytosis-associated genes. Early and late CMDMs were compared by using GSEA enrichment analysis. (**a**) Top 40 enriched GO BP terms are plotted in order of normalized enrichment scores. Dot size indicates the number of genes. Dot color indicates the adjusted p value (BH method). (**b**) GSEA enrichment plot of genes involved in cholesterol metabolism is shown. (**c**) Violin plots of indicated gene expression in Mo-Mac clusters 1-

4 are shown. (**d**) GSEA enrichment plot of genes involved in phagocytosis is shown. (**e**) GSEA enrichment plots of genes involved in lysosome and Rac1 GTPase are shown.

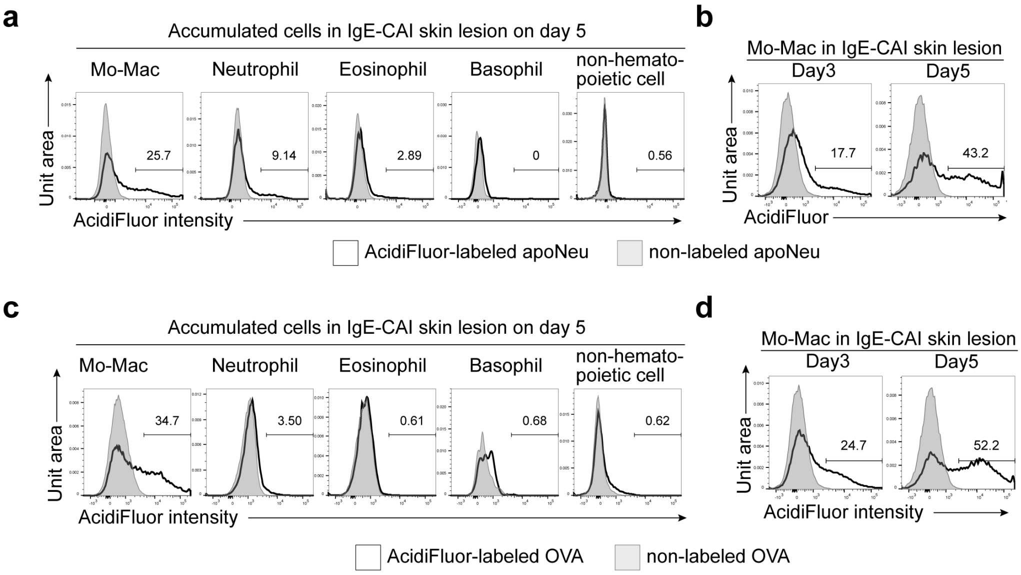

Supplementary Fig. 9. **Monocyte-macrophage lineage cells are the major cell types which phagocytose apoptotic cells and antigens in the IgE-CAI skin lesion.** WT mice were treated as in Fig. 1 to induce IgE-CAI. Apoptotic neutrophils (**a**-**b**) or OVA (**c**-**d**) labeled (open histograms) or unlabeled (shaded histograms) with AcidiFluor were intradermally administered to the skin lesion on day 5 post-challenge. Two hr after injection, ear skins were subjected to flow cytometric analysis. In **a** and **c**, histograms of AcidiFluor fluorescence in indicated cell types are shown. In **b** and **d**, WT mice were treated as in **a** and **c** on day 3 or 5 post-challenge and subjected to flow cytometric analysis 2 hr later. Data shown in a-d are representative of three independent experiments.

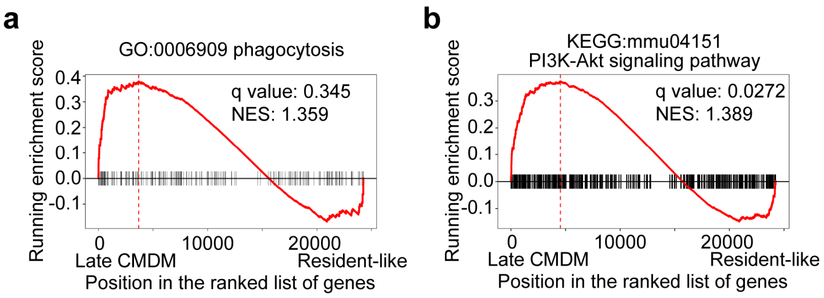

Supplementary Fig. 10. Phagocytosis-associated genes are rather enriched in late CMDMs compared to resident-like macrophages. (a-b) Late CMDMs and resident-like macrophages were compared by using GSEA enrichment analysis. GSEA enrichment plots of genes involved in phagocytosis (in a) and PI3K-Akt signaling pathway (in b) are shown.

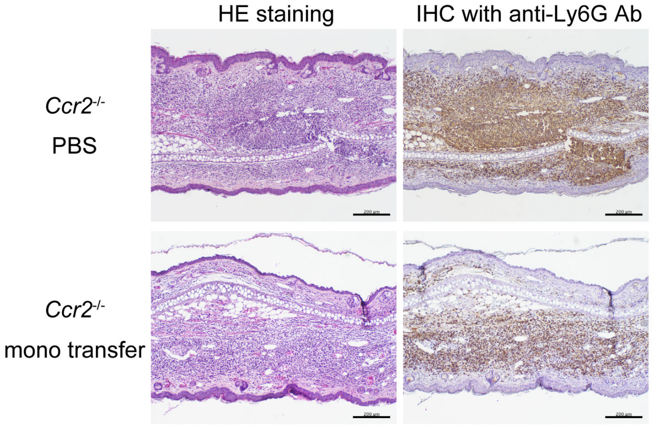

Supplementary Fig. 11. Adoptive transfer of monocytes reduced the formation of neutrophil-rich small aggregates of leukocytes in the IgE-CAI skin lesion of *Ccr2*-/- mice. *Ccr2*$^{-/-}$ mice were treated as in Fig. 1 to induce IgE-CAI. CD115^{+} monocytes isolated from the bone marrow of WT mice or control PBS were intravenously administered to mice five times on days 0, 1, 2, 3, and 4 post-challenge. Ear specimens collected on day 5 post-challenge were subjected to HE staining (left panels) or immunostaining with anti-Ly6G antibody (right panels). Bars indicate 200 µm. Data shown are representative of two independent experiments.

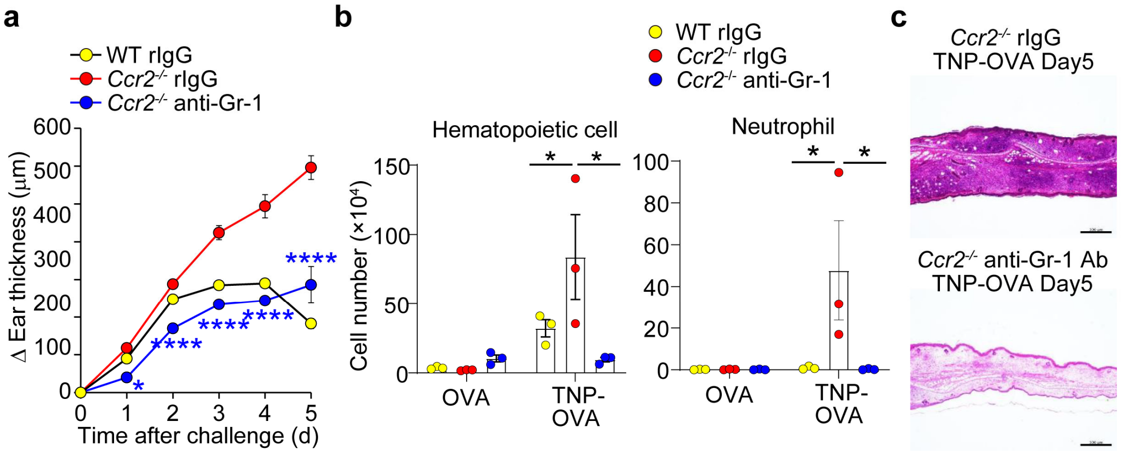

Supplementary Fig. 12. Administration of anti-Gr-1 antibody attenuates IgE-CAI in *Ccr2*$^{-/-}$ mice. WT and *Ccr2*$^{-/-}$ mice were treated as in Figure 1 to induce IgE-CAI. Neutrophil depletion antibody (anti-Gr-1) or control antibody (rIgG) was intraperitoneally administered to mice on days 0, 1, 2, 3, and 4 post-challenge. (a) Time course of ear swelling (Δ ear thickness) is shown (mean ± SEM, n=4, n=5, and n=5 biologically independent animals for WT rIgG group, *Ccr2*$^{-/-}$ rIgG group, and *Ccr2*$^{-/-}$ anti-Gr-1 group, respectively). **p*=0.0107 (for day 1), *****p*=7.56×10^{-5} (for day 2), *****p*=1.48×10^{-9} (for day 3), and *****p*<1.0×10^{-15} (for day 4 and day 5) measured by two-way ANOVA with Tukey's multiple comparison test. (b) The number of hematopoietic cells and neutrophils in the ear skin on day 5 is shown (mean ± SEM, n=3 biologically independent animals for each group). In the left panel, **p*=0.014 (*Ccr2*$^{-/-}$ rIgG vs. *Ccr2*$^{-/-}$ anti-Gr-1) measured by two-way ANOVA with Tukey's multiple comparison test. In the right panel **p*=0.0465 (WT rIgG vs. *Ccr2*$^{-/-}$ rIgG), **p*=0.0427 (*Ccr2*$^{-/-}$ rIgG vs. *Ccr2*$^{-/-}$ anti-Gr-1) measured by two-way ANOVA with Tukey's multiple comparison test. (**c**) HE stained ear specimens collected on day 5 are shown. Bars indicate 500 μm. Data shown are representative of two independent experiments. Source data are provided as a Source Data file.

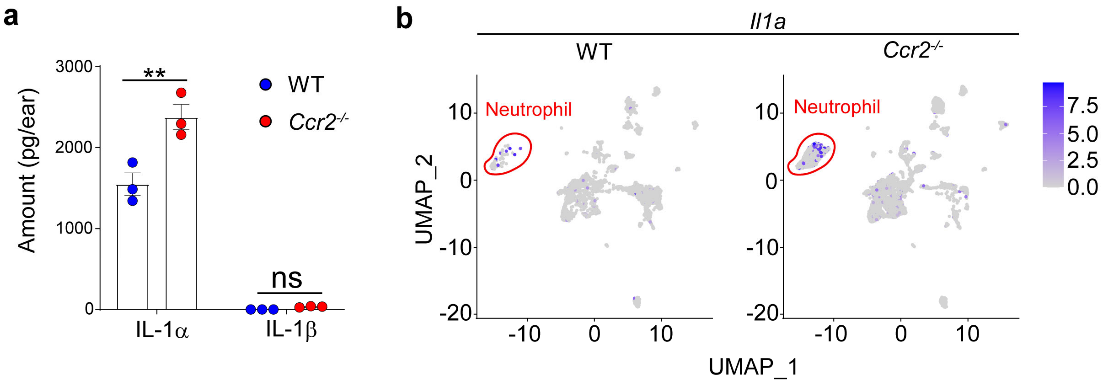

Supplementary Fig. 13. ***Il1a* mRNA is predominantly expressed in neutrophils in the IgE-CAI skin lesion of *Ccr2*$^{-/-}$ mice.** (a) WT and *Ccr2*$^{-/-}$ mice were treated as in Fig. 1 to induce IgE-CAI. The amounts of IL-1α and IL-1β in tissue homogenates collected from the IgE-CAI skin lesions on day 5 are shown (n=3 biologically independent animals for each group, mean ± SEM). ***p*=0.001 (for IL-1α), ns: *p*=0.9669 (for IL-1β) measured by two-way ANOVA with Tukey's multiple comparison test. (b) scRNA-seq datasets in Fig. 1f were analyzed. Feature plots showing the expression of *Il1a* in the IgE-CAI skin lesion of WT and *Ccr2*$^{-/-}$ mice are shown. Data shown in a are representative of two independent experiments. Source data are provided as a Source Data file.

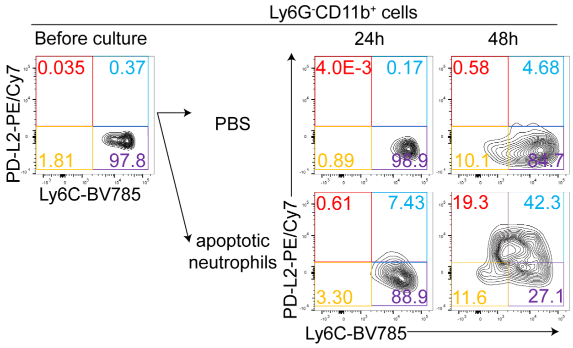

Supplementary Fig. 14. **Classical monocytes cultured with apoptotic neutrophils differentiate into early and late CMDMs.** Ly6C^{hi} classical monocytes isolated from the bone marrow of WT mice were incubated *ex vivo* with apoptotic neutrophils or control PBS. The surface expression of Ly6C and PD-L2 in monocytes before the culture (left) and after 24hr- or 48hr-incubation with BMBA supernatants (middle and right, respectively) are shown. Data shown are representative of three independent experiments.

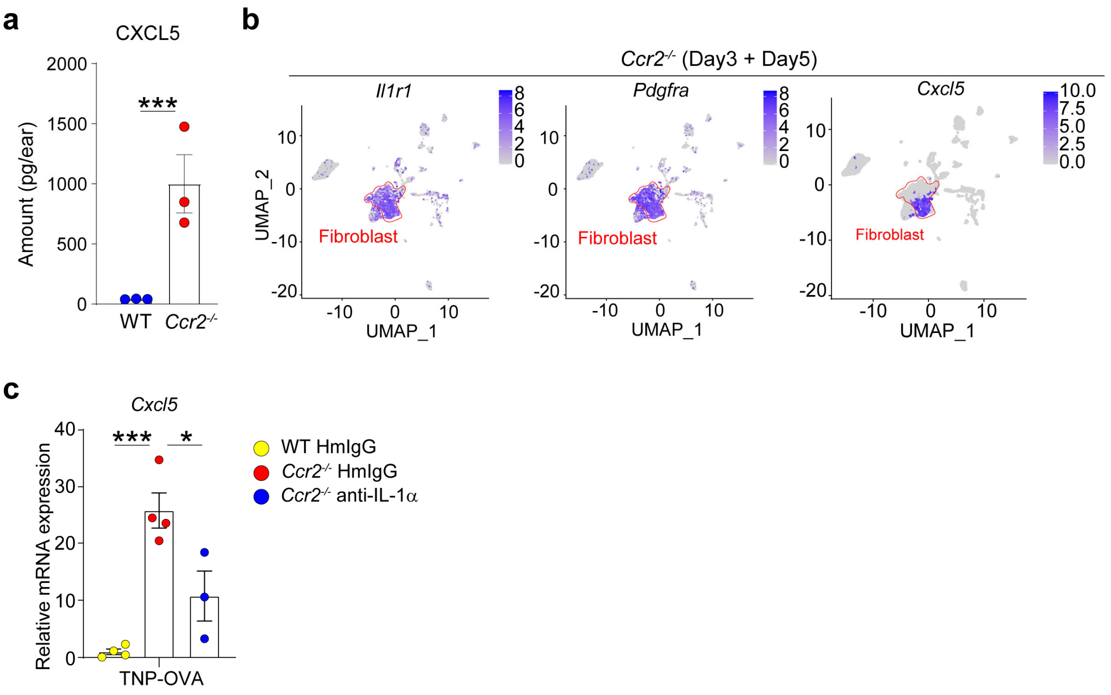

Supplementary Fig. 15. **IL-1α-stimulated fibroblasts produce neutrophil-attracting chemokine CXCL5 in *Ccr2*$^{-/-}$ mice, promoting the recruitment of neutrophils.** **(a)** WT and *Ccr2*$^{-/-}$ mice were treated as in Fig. 1 to induce IgE-CAI. The amount of CXCL5 in tissue homogenates collected from the IgE-CAI skin lesions on day 5 is shown (n=3 biologically independent animals for each group, mean ± SEM). *p=0.0168 measured by two-sided unpaired Student's t test. (b) scRNA-seq data shown in supplementary Figure S1a were re-analyzed. Feature plots displaying the expression of *Il1r1*, *Pdgfra, Cxcl5* genes in the skin lesion of *Ccr2*$^{-/-}$ mice are shown. (c) WT and *Ccr2*$^{-/-}$ mice were treated as in Figure 1 to induce IgE-CAI. IL-1α-neutralizing antibody or its isotype-matched control (HmIgG) was intraperitoneally administered to WT or *Ccr2*$^{-/-}$ mice on days 0, 1, 2, 3, and 4 post-challenge. Fibroblasts isolated from the IgE-CAI skin lesion were subjected to quantitative PCR analysis to assess gene expression of *Cxcl5* (mean ± SEM, n=4, n=4, and n=3 for WT HmIgG, *Ccr2*$^{-/-}$ HmIgG, and *Ccr2*$^{-/-}$ anti-IL-1α, respectively). ***p=0.0005 (WT HmIgG vs. *Ccr2*$^{-/-}$ HmIgG), *p=0.0157 (*Ccr2*$^{-/-}$ HmIgG vs. *Ccr2*$^{-/-}$ anti-IL-1α) measured by one-way ANOVA with Tukey's multiple comparison test. Data shown in a and c are representative of three independent experiments. Source data are provided as a Source Data file.
