# Supplementary material for: Single-cell transcriptomics identifies the differentiation trajectory from inflammatory monocytes to pro-resolving macrophages in a mouse skin allergy model
Source: Nat Commun. 2024 Feb 23;15:1666. doi: 10.1038/s41467-024-46148-4 (PMC10891131; doi:10.1038/s41467-024-46148-4)
Supplement: Supplementary file 3 — Reporting Summary [file 41467_2024_46148_MOESM3_ESM.pdf]

nature portfolio

Corresponding author(s): Kensuke Miyake

Last updated by author(s): Jan 30, 2024

Reporting Summary

Nature Portfolio wishes to improve the reproducibility of the work that we publish. This form provides structure for consistency and transparency in reporting. For further information on Nature Portfolio policies, see our Editorial Policies and the Editorial Policy Checklist.

Statistics

For all statistical analyses, confirm that the following items are present in the figure legend, table legend, main text, or Methods section.

n/a	Confirmed	
[ ]	[x]	The exact sample size (*n*) for each experimental group/condition, given as a discrete number and unit of measurement
[ ]	[x]	A statement on whether measurements were taken from distinct samples or whether the same sample was measured repeatedly
[ ]	[x]	The statistical test(s) used AND whether they are one- or two-sided<br>*Only common tests should be described solely by name; describe more complex techniques in the Methods section.*
[x]	[ ]	A description of all covariates tested
[ ]	[x]	A description of any assumptions or corrections, such as tests of normality and adjustment for multiple comparisons
[ ]	[x]	A full description of the statistical parameters including central tendency (e.g. means) or other basic estimates (e.g. regression coefficient) AND variation (e.g. standard deviation) or associated estimates of uncertainty (e.g. confidence intervals)
[ ]	[x]	For null hypothesis testing, the test statistic (e.g. *F*, *t*, *r*) with confidence intervals, effect sizes, degrees of freedom and *P* value noted<br>*Give P values as exact values whenever suitable.*
[x]	[ ]	For Bayesian analysis, information on the choice of priors and Markov chain Monte Carlo settings
[ ]	[x]	For hierarchical and complex designs, identification of the appropriate level for tests and full reporting of outcomes
[x]	[ ]	Estimates of effect sizes (e.g. Cohen's *d*, Pearson's *r*), indicating how they were calculated

Our web collection on statistics for biologists contains articles on many of the points above.

Software and code

Policy information about availability of computer code

Data collection	For bulk and single-cell RNA-seq analysis, cDNA libraries were sequenced by using Novaseq 6000 (Illumina) as described in Methods section.<br>No commercial, open source or custom code was used to collect data.
Data analysis	For scRNA-seq analysis, pair-end Fastq data were processed as follows: Adapter trimming, homopolymer trimming, and quality filitering of sequencing data was performed by using cutadapt. Associated cDNA reads were mapped to Ensembl RNA (GRCm38.p6, release-101) by using bowtie2. Then, cell barcode information of each read was added to the bowtie2-mapped BAM files, and read counts of each gene in each cell barcode were counted by using mawk. Resulted count data was converted to genes x cells matrix file and inflection threshold of the knee-plot was detected by using DropletUtils package in R. In addition, we further estimate background beads by using emptyDrops formula in DropletUtils package.<br>The resultant dataset was analyzed using R software package Seurat v4.0.4 in R 4.1.0. As quality control, doublets and cells with the mitochondrial gene proportion >20% were filtered out. The log-normalized gene counts were calculated using NormalizeData function (scale.factor = 1,000,000) and highly variable genes were defined by FindVariableFeatures function (selection.method="vst", nfeature=2000). Read counts were regressed out by the ScaleData function. Principal component analysis was performed on the variable genes, and principal components with their p-value <0.05 calculated by the jackstraw method were subjected to cell clustering and UMAP dimensional reduction. Differentially expressed genes were defined as those whose p-value, as calculated by the Wilcoxon rank sum test and adjusted by the Bonferroni method is <0.05 and whose log2FoldChange is >0.5 or <-0.5. GSEA was conducted by utilizing the R software package clusterProfiler v4.0.5. Pseudotime analysis was performed by utilizing slingshot v2.4.0 and tradeSeq. RNA velocity analysis was conducted by utilizing scVelo.<br><br>For bulk RNA-seq analysis, single-end Fastq files were processed as follows: Adapter trimming of sequencing data was performed by using cutadapt 4.1, and trimmed reads were mapped to reference RNA (build GRCm38 release-101) by using bowtie2-2.4.5 by the following

parameters: -p 8 -N 1 -L 16 –very-sensitive-local –seed 656565 –nofw. Then, read counts of each gene in each samples were counted by using awk, sort, and uniq -c commands. Resulted gene-expression count data was summarized by same Gene Symbol and all of the expression table was full-outer joined by Gene Symbol by using dplyr-1.0.7 package. Normalization of count data and DE analyses were performed by utilizing the R software package TCC v.1.32.0 63. PCA visualization was conducted by ggbiplot. Differentially expressed genes (DEGs) for each Mo-Mac subpopulation was defined as follows: DEGs for Ly6C+PD-L2- Mo-Mac were defined as genes significantly upregulated in Ly6ChiPD-L2lo Mo-Mac compared to Ly6ChiPD-L2hi and Ly6CloPD-L2hi Mo-Mac; DEGs for Ly6ChiPD-L2hi Mo-Mac were defined as genes significantly upregulated in Ly6ChiPD-L2hi Mo-Mac compared to Ly6ChiPD-L2lo and Ly6CloPD-L2hi Mo-Mac; DEGs for Ly6CloPD-L2hi Mo-Mac were defined as genes significantly upregulated in Ly6CloPD-L2hi Mo-Mac compared to Ly6ChiPD-L2hi and Ly6CloPD-L2lo Mo-Mac; DEGs for Ly6CloPD-L2lo Mo-Mac were defined as genes significantly upregulated in Ly6CloPD-L2lo Mo-Mac compared to Ly6CloPD-L2hi and Ly6ChiPD-L2lo Mo-Mac. Module scores for each Mo-Mac subpopulation was calculated by AddModuleScore function in Seurat v4.0.4.

The R codes for bulk and single-cell RNA-seq analyses are available at GitHub (https://github.com/KensukeMiyake/CMDM-paper).

For flow cytometric analysis, FlowJo ver 10.8.1 (BD Biosciences) was used.

For statistical analysis, GraphPad Prism (ver 7.0.3) was used.

For manuscripts utilizing custom algorithms or software that are central to the research but not yet described in published literature, software must be made available to editors and reviewers. We strongly encourage code deposition in a community repository (e.g. GitHub). See the Nature Portfolio guidelines for submitting code & software for further information.

Data

Policy information about availability of data

All manuscripts must include a data availability statement. This statement should provide the following information, where applicable:

- Accession codes, unique identifiers, or web links for publicly available datasets
- A description of any restrictions on data availability
- For clinical datasets or third party data, please ensure that the statement adheres to our policy

The scRNA-seq and bulk RNA-seq data generated in this study have been deposited in the NCBI Gene Expression Omnibus (GEO) database under accession code GSE221310 (https://www.ncbi.nlm.nih.gov/geo/query/acc.cgi?acc=GSE221310) and GSE245865 (https://www.ncbi.nlm.nih.gov/geo/query/acc.cgi?acc=GSE245865), respectively. For mapping of transcriptomic data, mouse EnsemblRNA (GRCm38, release-101; http://aug2020.archive.ensembl.org/Mus_musculus/Info/Index) was used. Source data are provided as a Source Data file.

Research involving human participants, their data, or biological material

Policy information about studies with human participants or human data. See also policy information about sex, gender (identity/presentation), and sexual orientation and race, ethnicity and racism.

Reporting on sex and gender	Research involving human participants, their data, or biological material was not conducted in this study.
Reporting on race, ethnicity, or other socially relevant groupings	Research involving human participants, their data, or biological material was not conducted in this study.
Population characteristics	Research involving human participants, their data, or biological material was not conducted in this study.
Recruitment	Research involving human participants, their data, or biological material was not conducted in this study.
Ethics oversight	Research involving human participants, their data, or biological material was not conducted in this study.

Note that full information on the approval of the study protocol must also be provided in the manuscript.

Field-specific reporting

Please select the one below that is the best fit for your research. If you are not sure, read the appropriate sections before making your selection.

☒ Life sciences ☐ Behavioural & social sciences ☐ Ecological, evolutionary & environmental sciences

For a reference copy of the document with all sections, see nature.com/documents/nr-reporting-summary-flat.pdf

Life sciences study design

All studies must disclose on these points even when the disclosure is negative.

Sample size	No statistical methods were used to determine sample size. According to the 3R principle, we chose the minimal replicate number sufficient to ascertain statistics by unpaired t-test, one-way ANOVA, or two-way ANOVA.
Data exclusions	No data were excluded from mice experiments.
Replication	All the experiments were successfully replicated at least three times.

Randomization	Animals used in this study were randomly assigned to their respective groups before the experiments were performed.
Blinding	Blinding was not achieved in this study due to requirements for cage identification and labeling for treatment purposes.

Reporting for specific materials, systems and methods

We require information from authors about some types of materials, experimental systems and methods used in many studies. Here, indicate whether each material, system or method listed is relevant to your study. If you are not sure if a list item applies to your research, read the appropriate section before selecting a response.

Materials & experimental systems

n/a	Involved in the study
☐	☒ Antibodies
☒	☐ Eukaryotic cell lines
☒	☐ Palaeontology and archaeology
☐	☒ Animals and other organisms
☒	☐ Clinical data
☒	☐ Dual use research of concern
☒	☐ Plants

Methods

n/a	Involved in the study
☒	☐ ChIP-seq
☐	☒ Flow cytometry
☒	☐ MRI-based neuroimaging

Antibodies

Antibodies used

The following antibodies were purchased from BioLegend:
Alexa Fluor 488-conjugated anti-CD45.2 (clone: 104, catalog #:109816, dilution 1:400, Lot: B234286)
APC-conjugated anti-CD200R3 (clone: Ba13, catalog#: 142208, dilution 1:400, Lot: B324365)
APC-conjugated anti-CD64 (clone: X54-5/7.1, catalog #:139306, dilution 1:400, Lot: B357450)
APC-Cy7-conjugated anti-Ly6G (clone: 1A8, catalog#: 127624, dilution 1:400, Lot: B333356)
APC/Fire 750-conjugated anti-Ly6G (clone: 1A8, catalog#: 127652, dilution 1:400, Lot: B316358)
FITC-conjugated anti-CD45 (clone: 30-F11, catalog#: 103108, dilution 1:400, Lot: B330230)
PacificBlue-conjugated anti-c-Kit (clone: 2B8, catalog#: 105820, dilution 1:400, Lot: B314152)
PacificBlue-conjugated anti-CD45.1 (clone: A20, catalog#: 110722, dilution 1:400, Lot: B191340)
BV421-conjugated PD-L2 (clone: TY25, catalog#: 107219, dilution 1:400, Lot: B291233)
BV421-conjugated anti-CD11b (clone: M1/70, catalog#: 101251, dilution 1:400, Lot: B336199)
BV510-conjugated anti-CD45.1 (clone: A20, catalog#: 110741, dilution 1:400, Lot: B312868)
BV605-conjugated anti-CD11b (clone: M1/70, catalog#: 101257, dilution 1:400, Lot: B350236)
BV711-conjugated anti-F4/80 (clone: BM8, catalog#: 123147, dilution 1:400, Lot: B370850)
BV785-conjugated anti-Ly6C (clone: HK1.4, catalog#: 128041, dilution 1:400, Lot: B371649)
PE-conjugated anti-PD-L2 (clone: TY25, catalog#: 107206, dilution 1:400, Lot:B146592)
PE-Cy7-conjugated anti-CD49b (clone: HMα2, catalog#: 103518, dilution 1:400, Lot: B348493)
PE-Cy7-conjugated anti-F4/80 (clone: BM8, catalog#:123114, dilution 1:400, Lot: B342137)
PE-Cy7-conjugated anti-PD-L2 (clone: TY25, catalog#: 107214, dilution 1:400, , Lot: B332455).
TruStain FcX PLUS antibody (anti-CD16/32 antibody, clone: S17011E, catalog#: 156604, dilution 1:200)

The following antibodies were purchased from BD Biosciences:
Alexa 647-conjugaed anti-Siglec-F (clone: E50-2440, catalog#: 562680, dilution 1:400, Lot: 0279640)
BV421-conjugated anti-Siglec-F (clone: E50-2440, catalog#: 562681, dilution 1:400, Lot: 1097452)
BV480-conjugated c-Kit (clone: 2B8, catalog#: 566074, dilution 1:400, Lot: 1013006).

The following antibodies were used in IHC:
Purified rat anti-Ly6G antibody (1 μg/mL; clone: 1A8, catalog#: 127602, Lot: B199551; BioLegend)
Purified rat IgG2a isotype control antibody (1 μg/mL; clone: RTK2758, catalog#: 400502, Lot: B186217; BioLegend)
Rabbit polyclonal anti-RIPK1 antibody (2.5 μg/mL, catalog#: NBP1-77077, Lot: 5389-1802; Novus Biologicals),
Rabbit IgG, polyclonal - Isotype Control (2.5 μg/mL, catalog#: ab37415, Lot: CR3219601-1; Abcam).

The following antibodies were used in vivo study:
Ultra-LEAF anti-mouse Ly6G antibody (clone: 1A8, catalog#: 127650, Lot: B218711; BioLegend).
anti-mouse Gr-1 antibody (clone: RB6-8C5; prepared in our laboratory from hybridoma originally produced by Robert L. Coffman, DNAX Research Institute)
control rat IgG (Rat Gamma Globulin) (catalog#: 012-000-002, Lot: 135648; Jackson ImmunoReseach)
InVivoMAb anti-mouse IL-1α (clone: ALF-161; BioXCell catalog#: BE0243)
InVivoMAb anti-mouse/rat IL-1β (clone: B122; BioXCell catalog#: BE0246)
InVivoMAb polyclonal Armenian hamster IgG (BioXCell catalog#: BE0091)
IgE mAb specific to 2,4,6-trinitrophenol (TNP) was prepared from hybridoma (IGELb4, purchased from ATCC; catalog#:TIB141) in our laboratory.

The following antibodies were used in scRNA-seq.
TotalSeq-A0311 anti-mouse Hashtag 11 Antibody (catalog #: 155821, dilution 1:400, Lot: B264842; BioLegend)
TotalSeq-A0312 anti-mouse Hashtag 12 Antibody (catalog #: 155823, dilution 1:400, Lot: B264843; BioLegend)
TotalSeq-A0313 anti-mouse Hashtag 13 Antibody (catalog #: 155825, dilution 1:400, Lot: B264845; BioLegend)
TotalSeq-A0314 anti-mouse Hashtag 14 Antibody (catalog #: 155827, dilution 1:400, Lot: B271401; BioLegend)

Validation

Antibodies were used according to the manufacturer's instructions, based on their provided methods of validation.
1) Alexa Fluor 488-conjugated anti-CD45.2 (clone: 104, catalog #:109816)
Verified Reactivity: Mouse, Application: FC - Quality tested
Each lot of this antibody is quality control tested by immunofluorescent staining with flow cytometric analysis. For flow cytometric staining, the suggested use of this reagent is ≤ 0.25 µg per 106 cells in 100 µl volume. It is recommended that the reagent be titrated for optimal performance for each application.
2) APC-conjugated anti-CD200R3 (clone: Ba13, catalog#: 142208)
Verified Reactivity: Mouse, Application: FC - Quality tested
Each lot of this antibody is quality control tested by immunofluorescent staining with flow cytometric analysis. For flow cytometric staining, the suggested use of this reagent is ≤0.25 µg per million cells in 100 µl volume. It is recommended that the reagent be titrated for optimal performance for each application.
3) APC-conjugated anti-CD64 (clone: X54-5/7.1, catalog #:139306)
Verified Reactivity: Mouse, Application: FC - Quality tested
Each lot of this antibody is quality control tested by immunofluorescent staining with flow cytometric analysis. For flow cytometric staining, the suggested use of this reagent is ≤1.0 µg per million cells in 100 µl volume. It is recommended that the reagent be titrated for optimal performance for each application.
4) APC-Cy7-conjugated anti-Ly6G (clone: 1A8, catalog#: 127624)
Verified Reactivity: Mouse, Application: FC - Quality tested
Each lot of this antibody is quality control tested by immunofluorescent staining with flow cytometric analysis. For flow cytometric staining, the suggested use of this reagent is ≤0.25 µg per million cells in 100 µl volume. It is recommended that the reagent be titrated for optimal performance for each application.
5) APC/Fire 750-conjugated anti-Ly6G (clone: 1A8, catalog#: 127652)
Verified Reactivity: Mouse, Application: FC - Quality tested
Each lot of this antibody is quality control tested by immunofluorescent staining with flow cytometric analysis. For flow cytometric staining, the suggested use of this reagent is ≤0.5 µg per million cells in 100 µl volume. It is recommended that the reagent be titrated for optimal performance for each application.
6) FITC-conjugated anti-CD45 (clone: 30-F11, catalog#: 103108)]
Verified Reactivity: Mouse, Application: FC - Quality tested
Each lot of this antibody is quality control tested by immunofluorescent staining with flow cytometric analysis. For flow cytometric staining, the suggested use of this reagent is ≤ 0.25 µg per 106 cells in 100 µl volume. It is recommended that the reagent be titrated for optimal performance for each application.
7) PacificBlue-conjugated anti-c-Kit (clone: 2B8, catalog#: 105820)
Verified Reactivity: Mouse, Application: FC - Quality tested
Each lot of this antibody is quality control tested by immunofluorescent staining with flow cytometric analysis. The suggested use of this reagent is ≤ 1.0 µg per 106 cells in 100 µl volume. It is highly recommended that the reagent be titrated for optimal performance for each application.
8) PacificBlue-conjugated anti-CD45.1 (clone: A20, catalog#: 110722)
Verified Reactivity: Mouse, Application: FC - Quality tested
Each lot of this antibody is quality control tested by immunofluorescent staining with flow cytometric analysis. The suggested use of this reagent is ≤ 1.0 µg per 106 cells in 100 µl volume. It is highly recommended that the reagent be titrated for optimal performance for each application.
9) BV421-conjugated PD-L2 (clone: TY25, catalog#: 107219)
Verified Reactivity: Mouse, Application: FC - Quality tested
Each lot of this antibody is quality control tested by immunofluorescent staining with flow cytometric analysis. For flow cytometric staining, the suggested use of this reagent is ≤ 0.25 µg per million cells in 100 µl volume. It is recommended that the reagent be titrated for optimal performance for each application.
10) BV421-conjugated anti-CD11b (clone: M1/70, catalog#: 101251)
Verified Reactivity: Mouse, Human, Cynomolgus, Rhesus, Application: FC - Quality tested
Each lot of this antibody is quality control tested by immunofluorescent staining with flow cytometric analysis. For flow cytometric staining using the µg size, the suggested use of this reagent is ≤ 0.25 µg per million cells in 100 µL volume. For flow cytometric staining using the µL sizes, the suggested use of this reagent is 5 µL per million cells in 100 µL staining volume or 5 µL per 100 µL of whole blood. It is recommended that the reagent be titrated for optimal performance for each application.
11) BV510-conjugated anti-CD45.1 (clone: A20, catalog#: 110741)
Verified Reactivity: Mouse, Application: FC - Quality tested
Each lot of this antibody is quality control tested by immunofluorescent staining with flow cytometric analysis. For flow cytometric staining, the suggested use of this reagent is ≤0.5 µg per million cells in 100 µl volume. It is recommended that the reagent be titrated for optimal performance for each application.
12) BV605-conjugated anti-CD11b (clone: M1/70, catalog#: 101257)
Verified Reactivity: Mouse, Human, Cynomolgus, Rhesus, Application: FC - Quality tested
Each lot of this antibody is quality control tested by immunofluorescent staining with flow cytometric analysis. For flow cytometric staining using the µg size, the suggested use of this reagent is ≤0.25 µg per million cells in 100 µl volume. For flow cytometric staining using the µl size, the suggested use of this reagent is 5 µl per million cells in 100 µl staining volume or 5 µl per 100 µl of whole blood. It is recommended that the reagent be titrated for optimal performance for each application.
13) BV711-conjugated anti-F4/80 (clone: BM8, catalog#: 123147)

Verified Reactivity: Mouse, Application: FC - Quality tested
Each lot of this antibody is quality control tested by immunofluorescent staining with flow cytometric analysis. For flow cytometric staining, the suggested use of this reagent is ≤0.5 µg per million cells in 100 µl volume. It is recommended that the reagent be titrated for optimal performance for each application.
14) BV785-conjugated anti-Ly6C (clone: HK1.4, catalog#: 128041)
Verified Reactivity: Mouse, Application: FC - Quality tested
Each lot of this antibody is quality control tested by immunofluorescent staining with flow cytometric analysis. For flow cytometric staining, the suggested use of this reagent is ≤0.125 µg per million cells in 100 µl volume. It is recommended that the reagent be titrated for optimal performance for each application.
15) PE-conjugated anti-PD-L2 (clone: TY25, catalog#: 107206)
Verified Reactivity: Mouse, Application: FC - Quality tested
Each lot of this antibody is quality control tested by immunofluorescent staining with flow cytometric analysis. For flow cytometric staining, the suggested use of this reagent is ≤1.0 µg per million cells in 100 µl volume. It is recommended that the reagent be titrated for optimal performance for each application.
16) PE-Cy7-conjugated anti-CD49b (clone: HMα2, catalog#: 103518)
Verified Reactivity: Mouse, Application: FC - Quality tested
Each lot of this antibody is quality control tested by immunofluorescent staining with flow cytometric analysis. For flow cytometric staining, the suggested use of this reagent is ≤0.125 µg per million cells in 100 µl volume. It is recommended that the reagent be titrated for optimal performance for each application.
17) PE-Cy7-conjugated anti-F4/80 (clone: BM8, catalog#:123114)
Verified Reactivity: Mouse, Application: FC - Quality tested
Each lot of this antibody is quality control tested by immunofluorescent staining with flow cytometric analysis. For flow cytometric staining, the suggested use of this reagent is ≤0.25 µg per million cells in 100 µl volume. It is recommended that the reagent be titrated for optimal performance for each application.
18) PE-Cy7-conjugated anti-PD-L2 (clone: TY25, catalog#: 107214, dilution 1:400, , Lot: B332455).
Verified Reactivity: Mouse, Application: FC - Quality tested
Each lot of this antibody is quality control tested by immunofluorescent staining with flow cytometric analysis. For flow cytometric staining, the suggested use of this reagent is = 0.25 µg per million cells in 100 µl volume. It is recommended that the reagent be titrated for optimal performance for each application.
19) BD Pharmingen Alexa Fluor 647 Rat Anti-Mouse Siglec-F (clone: E50-2440, catalog#: 562680)
Reactivity: Mouse (QC Testing), Application: Flow cytometry (Routinely Tested)
20) BD Horizon BV421 Rat Anti-Mouse Siglec-F (clone: E50-2440, catalog#: 562681, dilution 1:400, Lot: 1097452)
Reactivity: Mouse (QC Testing), Application: Flow cytometry (Routinely Tested)
21) BD Horizon BV480 Rat Anti-Mouse CD117 (clone: 2B8, catalog#: 566074).
Reactivity: Mouse (QC Testing), Application: Flow cytometry (Routinely Tested)
22)Purified rat anti-Ly6G antibody (catalog#: 127602, BioLegend)
Verified Reactivity: Mouse, Application: FC - Quality tested, IHC-F - Verified, IHC - Reported in the literature, not verified in house
Each lot of this antibody is quality control tested by immunofluorescent staining with flow cytometric analysis. For flow cytometric staining, the suggested use of this reagent is ≤ 0.25 µg per 106 cells in 100 µl volume. It is recommended that the reagent be titrated for optimal performance for each application.
23)Purified rat IgG2a isotype control antibody (catalog#: 400502, BioLegend)
Application:FC - Quality tested, ChIP - Verified, ICFC, WB, IP, ICC, IHC, FA - Reported in the literature, not verified in house
Each lot of this antibody is quality control tested by immunofluorescent staining with flow cytometric analysis as negative control, and the purity is greater than 95% by SDS-PAGE. Use at concentrations comparable to those of the specific antibody of interest.
24) Rabbit polyclonal anti-RIPK1 antibody (2.5 µg/mL, catalog#: NBP1-77077, Lot: 5389-1802; Novus Biologicals)
Reactivity: Hu, Mu, Rt, Applications WB, ELISA, ICC/IF, IHC, KD
Validated by Biological Strategies.
25)Rabbit IgG, polyclonal - Isotype Control (2.5 µg/mL, catalog#: ab37415, Lot: CR3219601-1; Abcam).
Application: IHC-P, ICC/IF, ELISA, Flow Cyt, ChIP/Chip
https://www.abcam.co.jp/products/primary-antibodies/rabbit-igg-polyclonal-isotype-control-ab37415.html
26) Ultra-LEAF anti-mouse Ly6G antibody
Application: FC - Quality tested; Depletion, IHC - Reported in the literature, not verified in house
Each lot of this antibody is quality control tested by immunofluorescent staining with flow cytometric analysis. For flow cytometric staining, the suggested use of this reagent is ≤0.25 µg per million cells in 100 µl volume or 100 µl of whole blood. It is recommended that the reagent be titrated for optimal performance for each application.
27) anti-mouse Gr-1 antibody (clone: RB6-8C5; prepared in our laboratory from hybridoma) was validated in our laboratory.
28) control rat IgG (Rat Gamma Globulin) (catalog#: 012-000-002, Jackson ImmunoReseach)
Gamma globulins are purified from non-immunized animal serums by salt fractionation, ion-exchange chromatography and gel filtration. Gamma globulins are an inexpensive source of IgG with only trace amounts of other immunoglobulins and/or non-immunoglobulin serum proteins. Gamma globulins are supplied as sterile liquids in phosphate buffer without stabilizers or preservatives.
29) InVivoMAb anti-mouse IL-1α (clone: ALF-161; BioXCell catalog#: BE0243)
Reported Applications: in vivo IL-1α neutralization, in vitro IL-1α neutralization
https://bioxcell.com/invivomab-anti-mouse-il-1a#references
30) InVivoMAb anti-mouse/rat IL-1β (clone: B122; BioXCell catalog#: BE0246)
Reported Applications: in vivo IL-1β neutralization, in vitro IL-1β neutralization,ELISA
https://bioxcell.com/invivomab-anti-mouse-rat-il-1b
31) InVivoMAb polyclonal Armenian hamster IgG (BioXCell catalog#: BE0091)
https://bioxcell.com/invivomab-polyclonal-armenian-hamster-igg-be0091
32) Validation of TNP-specific IgE antibody (IGELb4, ATCC-TIB141) was conducted in our laboratory.

33) TotalSeq-A0311 anti-mouse Hashtag 11 Antibody (catalog #: 155821, dilution 1:400, Lot: B264842; BioLegend)
TotalSeq-A0312 anti-mouse Hashtag 12 Antibody (catalog #: 155823, dilution 1:400, Lot: B264843; BioLegend)
TotalSeq-A0313 anti-mouse Hashtag 13 Antibody (catalog #: 155825, dilution 1:400, Lot: B264845; BioLegend)
TotalSeq-A0314 anti-mouse Hashtag 14 Antibody (catalog #: 155827, dilution 1:400, Lot: B271401; BioLegend)
Verified Reactivity: Mouse, Application: PG - Quality tested
Each lot of this antibody is quality control tested by immunofluorescent staining with flow cytometric analysis and the oligomer sequence is confirmed by sequencing. TotalSeq™-A antibodies are compatible with 10x Genomics Single Cell Gene Expression Solutions.
To maximize performance, it is strongly recommended that the reagent be titrated for each application, and that you centrifuge the antibody dilution before adding to the cells at 14,000xg at 2 - 8°C for 10 minutes. Carefully pipette out the liquid avoiding the bottom of the tube and add to the cell suspension. For Proteogenomics analysis, the suggested starting amount of this reagent for titration is ≤ 1.0 µg per million cells in 100 µL volume. Refer to the corresponding TotalSeq™ protocol for specific staining instructions.

Animals and other research organisms

Policy information about studies involving animals; ARRIVE guidelines recommended for reporting animal research, and Sex and Gender in Research

Laboratory animals	BALB/c (BALB/cCrSlc) and C57BL/6J (C57BL/6JmsSlc) mice were purchased from Sankyo Labo Service Corporation, Inc, Japan. CD45.1 congenic C57BL/6 (B6.SJL-PtprcaPepcb/BoyJ) mice, Cx3cr1Cre C57BL/6 (B6J.B6N(Cg)-Cx3cr1tm1.1(cre)Jung/J) mice 46 and Il4ra-/- BALB/c (BALB/c-Il4ratm1Sz) mice 47 were purchased from the Jackson Laboratory. Il4rafl (Il4ratm2Fbb) mice 48 were previously established. Cx3cr1Cre and Il4rafl mice were cross-bred in our laboratory to establish Cx3cr1Cre Il4rafl mice that are deficient for IL-4 receptor only in Mo-Mac lineage cells. Ccr2-/- BALB/c (Ccr2tm1Mae) mice were kindly provided from W.A. Kuziel (External Scientific Affairs, Daiichi Sankyo Group, Edison, NJ) and N. Mukaida (Kanazawa University). Ccr2-/- C57BL/6 mice were generated by N. Mukaida (Kanazawa University) by backcrossing of Ccr2-/- BALB/c mice to C57BL/6 strain. Mice were maintained under specific pathogen-free conditions in our animal facilities. Animal rooms are maintained at 22±3°C, with a 12h:12h light-dark cycle, and humidity is maintained between 30% and 70%. Animals are housed in individually ventilated cages (Innovive Caging System) and are fed with natural ingredient chow diet ad libitum (Japan CLEA: Rodent Diet CE-2). Cages are bedded with cloth material (Japan SLC: Q-pura chip). Animals are group housed whenever possible. 7-12 week-old male mice were used in the study. At the end of all experiments, mice were euthanized by CO2 inhalation.
Wild animals	No wild animals were used in the study.
Reporting on sex	Data presented here is based on male mice, but the findings presented in this study were not restricted to male mice.
Field-collected samples	No field collected samples were used in the study.
Ethics oversight	All animal studies were approved by the Institutional Animal Care and Use Committee of Tokyo Medical and Dental University (No. A2022-023C2).

Note that full information on the approval of the study protocol must also be provided in the manuscript.

Plants

Seed stocks	n/a
Novel plant genotypes	n/a
Authentication	n/a

Flow Cytometry

Plots

Confirm that:

- [x] The axis labels state the marker and fluorochrome used (e.g. CD4-FITC).
- [x] The axis scales are clearly visible. Include numbers along axes only for bottom left plot of group (a 'group' is an analysis of identical markers).
- [x] All plots are contour plots with outliers or pseudocolor plots.
- [x] A numerical value for number of cells or percentage (with statistics) is provided.

Methodology

Sample preparation	For flow cytometric analyses, single cell suspensions were prepared from the ear skin by treating excised ears with collagenase (125 U/mL, Wako) in RPMI complete medium at 37°C for 2 h, followed by depletion of red blood cells. After pre-incubation with TruStain FcX PLUS antibody (anti-CD16/32 antibody; BioLegend) and normal rat serum (Merck Millipore) on ice for 10 min to prevent the non-specific binding of irrelevant Abs, cells were stained with indicated combination of Abs, and analyzed with FACSLyric (BD Biosciences) or sorted with FACSAriaIII (BD Biosciences).
Instrument	Flow-cytometric analysis was perfoemed using BD FACS Lyric flow cytometer (BD Biosciences). Cell sorting was performed using BD FACS AriaIII (BD Biosciences).
Software	Flow cytometry data were analyzed using FlowJo software (version 10.8.1, BD Biosciences)
Cell population abundance	Purity for all cell populations were confirmed to be greater than 95%.
Gating strategy	Each cell lineage was identified as follows: neutrophils (CD45+ Ly6G+ Siglec-F-), eosinophils (CD45+ Ly6Gint Siglec-F+), basophils (CD45int *c*-Kit- CD49b+ CD200R3+), Mo-Macs (CD45+ Ly6G- Siglec-F- CD11b+ F4/80+), and non-hematopoietic cells (CD45-).

☒ Tick this box to confirm that a figure exemplifying the gating strategy is provided in the Supplementary Information.
